# Skin-derived vascularized lymph node transfer combining with liposuction in stages for bilateral lower extremity lymphedema: a case report

**DOI:** 10.3389/fonc.2025.1677136

**Published:** 2025-10-13

**Authors:** Wenjie Pan, Liangliang Wang, Miaomiao Wei, Xiangkui Wu, Hai Li, Bihua Wu, Shune Xiao, Chengliang Deng

**Affiliations:** ^1^ Department of Burns and Plastic Surgery, Affiliated Hospital of Zunyi Medical University, Zunyi, Guizhou, China; ^2^ The Collaborative Innovation Center of Tissue Damage Repair and Regeneration Medicine, Zunyi Medical University, Zunyi, Guizhou, China

**Keywords:** lymphedema, bilateral, vascularized lymph node transfer, liposuction, surgery

## Abstract

Bilateral lower extremity lymphedema (LEL) significantly impairs patients’ quality of life. A 66-year-old female patient, who developed bilateral LEL following cervical cancer surgery (classified as International Society of Lymphology stage II), presented to our hospital. In a two-stage surgical approach, we employed two distinct lymph node transfer techniques: (1) transplantation of a 7 × 2 cm vascularized lymph node flap harvested from the right supraclavicular region to the left popliteal fossa, and (2) transplantation of a 9 × 3 cm skin-derived lymph node flap obtained from the submental area to the right popliteal fossa. Both procedures were combined with liposuction. Postoperative management included compression therapy, with no complications observed during the recovery period. The patient demonstrated significant improvement in quality-of-life measures. Lymphoscintigraphic imaging at the 12-month follow-up revealed improvement in lymphatic function, showing volume reductions of 2129.28 mL (left lower limb) and 1699.65 mL (right lower limb). No recurrence of swelling was reported during the 30-month follow-up period. This case suggests that a two-stage approach combining vascularized lymph node transfer (VLNT) with liposuction may represent an effective treatment strategy for bilateral lower extremity lymphedema.

## Introduction

Bilateral lower extremity lymphedema (LEL) represents a relatively uncommon yet potentially severe complication that may develop following pelvic lymph node dissection and subsequent radiation therapy (1). The management of this condition poses significant clinical challenges, with primary therapeutic objectives focusing on lymphatic flow restoration and secondary complication mitigation (2). In advanced lymphedema cases, vascularized lymph node transfer (VLNT) has been shown to enhance lymphatic circulation through two primary mechanisms: (1) establishing new lymphatic pathways via lymphangiogenesis between transferred lymph nodes and surrounding tissues, and (2) utilizing native lymphovenous shunts within the transferred lymph nodes (3, 4). Advanced-stage lymphedema is characterized by substantial fibrofatty tissue deposition in affected limbs. Consequently, optimal therapeutic outcomes require not only lymphatic flow improvement through VLNT but also excess fat removal via liposuction (5). Current evidence indicates that optimal management of advanced lymphedema requires combining physiological reconstructive surgery with debulking procedures (5, 6). Vascularized lymph node transfers can be harvested from various donor sites, including deeper omental tissue and superficial regions such as the groin, axilla, lateral thoracic area, cervical region, submental area, and omental area (7). While omental lymph node transfer combined with liposuction has been reported for bilateral LEL treatment (8, 9), this approach requires the assistance of a general surgeon, potentially limiting its widespread adoption. Supraclavicular and submental lymph node transfer are now commonly used because of the low risk of iatrogenic lymphedema. Our research team has previously demonstrated promising surgical outcomes using VLNT combined with liposuction for unilateral lower limb lymphedema management (10). However, there remains a significant gap in evidence regarding the application of VLNT combined with liposuction for guiding surgical strategy evaluation and selection in bilateral LEL cases.

This report presents successful clinical outcomes of a novel two-stage surgical approach combining supraclavicular and submental VLNT with liposuction for bilateral LEL management.

## Case report

**Figure 1:**
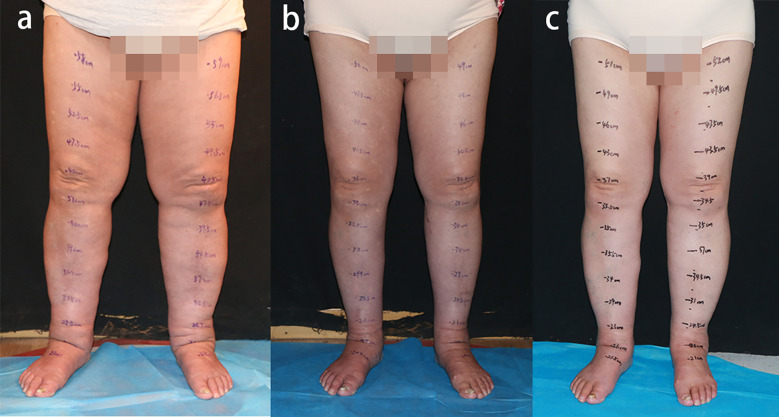
Preoperative and postoperative photographs of lower extremities. **(a)** Preoperative view. **(b)** Twelve-month postoperative result. **(c)** Thirty-month postoperative status. Significant reduction in circumference and volume of bilateral lower extremities was achieved, with patient-reported symptomatic improvement and absence of complications.

**Figure 2:**
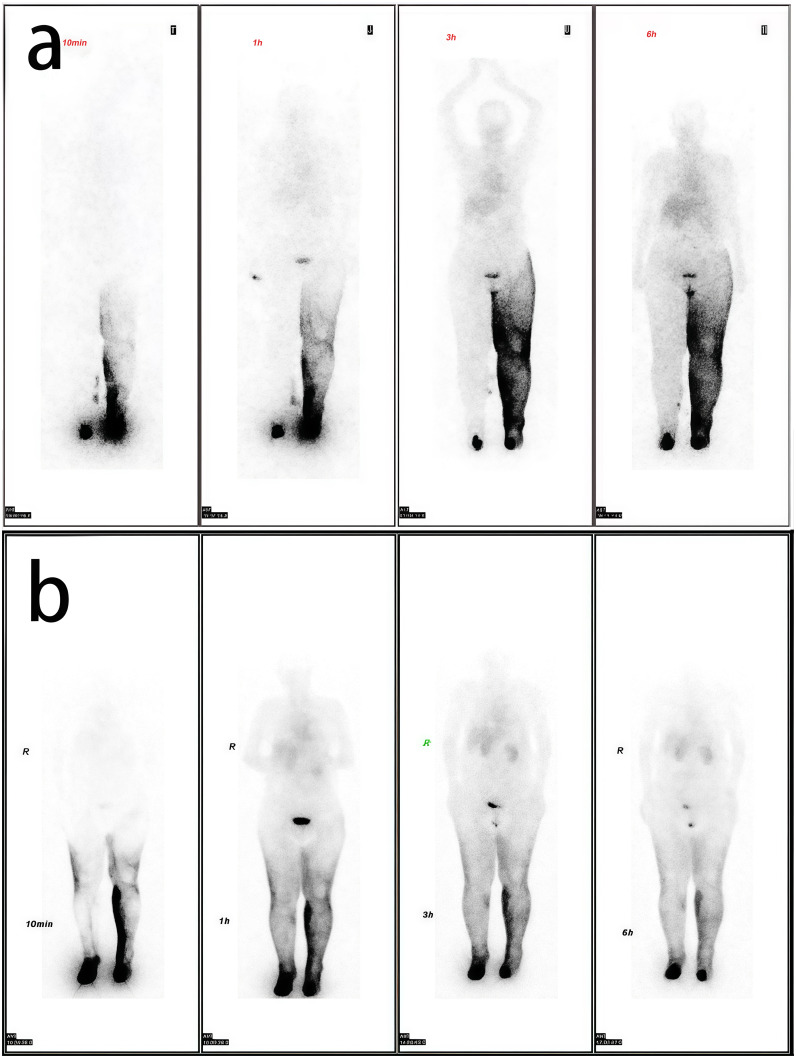
Comparative lymphoscintigraphy. **(a)** Preoperative study showing complete right-sided and partial left-sided lymphatic obstruction. **(b)** Postoperative study demonstrating restored bilateral drainage.

A 66-year-old female patient with a two-year history of cervical cancer underwent radical hysterectomy with subsequent adjuvant radiotherapy. Following an 11-month latency period, the patient developed bilateral lower extremity swelling accompanied by a tightness sensation. Progressive symptoms including erythema, swelling, and increasing pain subsequently developed, resulting in mobility impairment and significant discomfort that necessitated hospital consultation (**Figure 1**). Despite attempting alternative management strategies including traditional Chinese medicine and acupuncture, the patient showed no significant clinical improvement. Lymphoscintigraphic imaging revealed bilateral lower extremity thickening, with diffuse tracer distribution in the left lower limb soft tissues. The right lower extremity demonstrated non-visualization on imaging (**Figure 2**). This patient presented with bilateral lower-limb lymphedema classified as Stage III per the International Society of Lymphology (ISL) criteria. According to the Taiwan Lymphoscintigraphy staging criteria, the left limb was categorized as Stage P-3 and the right limb as Stage T-5. Based on Taiwan lymphoscintigraphy staging criteria (11), the right lower extremity exhibited more severe involvement compared to the left. Interestingly, the patient reported greater functional impairment and more pronounced swelling in the left lower extremity. Considering the patient’s clinical presentation, surgical risks, and prolonged operative time associated with simultaneous bilateral procedures, we implemented a two-stage surgical strategy. The initial stage involved left limb treatment through supraclavicular VLNT combined with liposuction. Following a three-month recovery period, the patient underwent second-stage surgical intervention involving submental VLNT combined with liposuction for right limb treatment.

**Figure 3:**
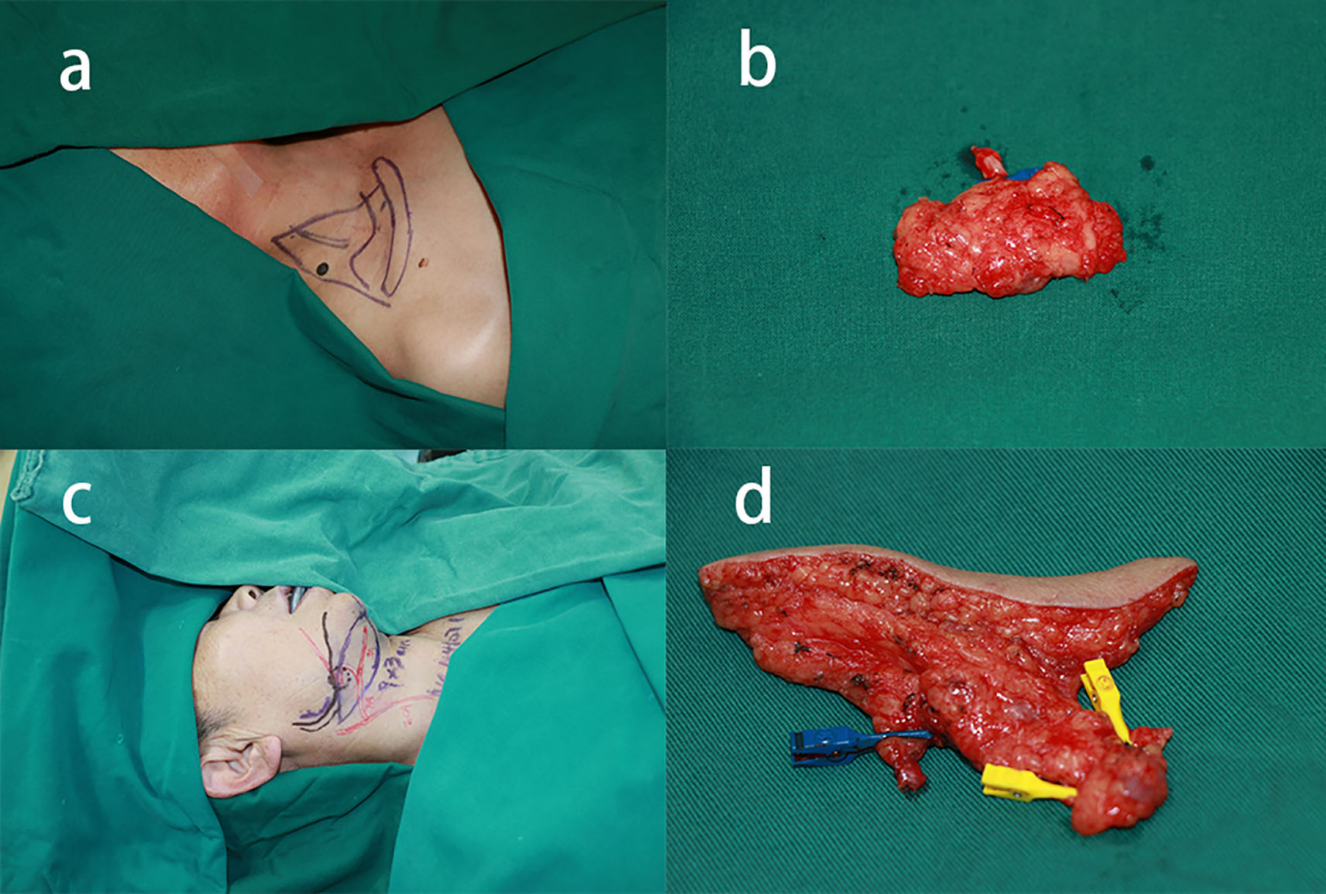
Two-stage surgical approach (supraclavicular VLNT + submental VLNT with liposuction). **(a)** Supraclavicular donor site design. **(b)** Harvested supraclavicular lymph node flap. **(c)** Submental donor site marking. **(d)** Harvested submental lymph node flap.

Building upon our previously established surgical protocol (10), we successfully harvested a supraclavicular lymph node flap (**Figure 3**). The harvested lymph node flap, situated inferior to the omohyoid muscle, incorporated the transverse cervical artery with its accompanying veins and associated deep lymph nodes. The lymph node flap was subsequently transferred and anastomosed end-to-end to the left medial sural artery. Concurrently, liposuction was performed on the left thigh, removing approximately 1000 mL of adipose tissue following tumescent solution infiltration. Following confirmation of anastomotic patency, we placed a negative pressure drain and applied compressive bandaging to the thigh. The procedure was completed without intraoperative complications or significant bleeding. Postoperative management included drain removal on day 3 and discontinuation of prophylactic antibiotic therapy. Postoperative compression therapy for both the lower leg and thigh is crucial for maintaining liposuction-induced volume reduction (12). Our compression protocol involved non-compressive gauze bandaging of the lower legs in the early postoperative period to ensure flap survival, while immediate compression garment application was implemented for the thigh liposuction areas. At one week postoperatively, specialized lymphedema elastic bandages were applied to achieve standardized gradient compression (13). Following six months of compressive bandaging, the patient was transitioned to custom-fitted compression stockings for long-term management.

Three months following the initial procedure, the patient returned for a second-stage operation targeting the right lower extremity, utilizing the right submental region as the donor site (**Figure 3**). Surgical dissection identified the facial artery and accompanying veins, enabling harvest of lymph nodes with surrounding fascial tissue as a vascularized free flap, using the facial artery as the vascular pedicle. The harvested flap was subsequently transferred to the distal right lower extremity. Microsurgical end-to-end anastomosis was performed between the facial artery and right medial sural artery, with corresponding venous anastomoses completed. Consistent with the previous procedure, 1000 mL of adipose tissue was removed through liposuction of the right thigh. Postoperative management mirrored the initial procedure, with the patient achieving uncomplicated recovery and discharge on postoperative day 6.

At the 12-month follow-up, significant volume reduction in both lower limbs was observed based on the calculation using the truncated cone formula (14). The measured reductions were 2129.28 mL (left) and 1699.65 mL (right). This reduction includes the volume of fat removed by liposuction (**Figure 1**). Follow-up lymphoscintigraphic imaging revealed improvement in bilateral lymphatic drainage (**Figure 2**).

**Figure 4:**
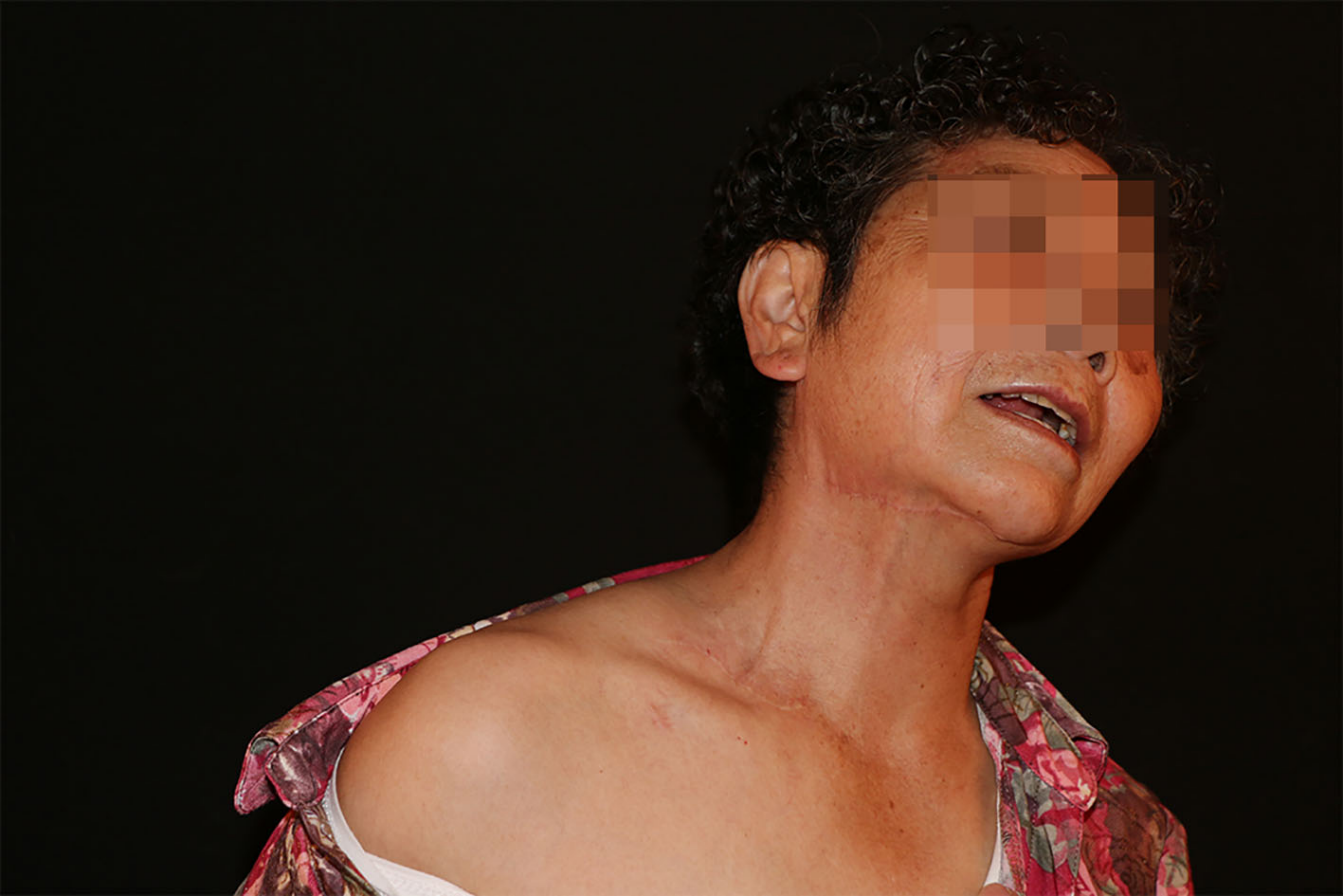
Thirty-month postoperative donor site appearance showing inconspicuous scarring without iatrogenic lymphedema.

At the 30-month postoperative follow-up, the patient presented for clinical evaluation. Donor site evaluation revealed minimal scarring with no evidence of iatrogenic lymphedema or surgical complications (**Figure 4**). Circumferential measurements confirmed sustained therapeutic efficacy without lymphedema recurrence (**Figure 1**). The Lymphedema Quality of Life (LYMQOL) questionnaire serves as a validated instrument for comprehensive quality-of-life assessment. This validated tool evaluates four domains: symptom, emotional well-being, functionality, and appearance, providing a quantitative assessment of patient-reported outcomes (15). Comparative LYMQOL assessment demonstrated improvement from a preoperative score of 6 to a postoperative score of 8, indicating enhanced quality-of-life measures.

## Discussion

To our knowledge, this represents the first documented case utilizing combined skin-derived VLNT and liposuction for bilateral LEL management. To minimize thoracic duct injury risk, we implemented a combined surgical approach incorporating right supraclavicular and submental lymph node transfers with concurrent liposuction for bilateral LEL treatment. Anatomical investigations have confirmed consistent anatomical patterns and predictable lymph node distribution in both the right supraclavicular region and submental area (16, 17). Maintenance of lymph node vascularization is essential for graft viability, as transferred lymphatic tissue demonstrates rapid integration and regenerative capacity within the recipient’s lymphatic system (18, 19).

At the 12-month postoperative follow-up, both lower extremities demonstrated significant volume reduction, with lymphographic imaging confirming improved lymphatic drainage patterns. VLNT is believed to promote lymphangiogenesis and either facilitate the transport of lymphatic fluid into proximal lymphatic channels or function as a pump to direct lymphatic fluid into the venous circulation (3, 20, 21).

The therapeutic effects of liposuction on edema reduction and imaging improvement may be mediated through multiple mechanisms: (1) enhanced peripheral blood flow, (2) reduced lymph production secondary to decreased subcutaneous adipose tissue volume, (3) formation of lymphovenous connections, and (4) development of fascial openings facilitating superficial-to-deep lymphatic drainage (22).

Some clinical studies have demonstrated the therapeutic efficacy of combined VLNT and liposuction for lymphedema management (5, 10, 23).

The combined approach offers substantial benefits, including rapid limb volume reduction accompanied by significant functional and psychological improvements. Liposuction effectively eliminates excess adipose tissue and fibrotic deposits through minimal skin incisions. Postoperative compression therapy plays a critical role in maintaining liposuction outcomes by promoting hypertrophied skin contraction and preventing fluid accumulation (18). However, whether liposuction can restore lymphatic return function remains controversial. Liposuction should be combined with physiological procedures to reduce the recurrence of secondary lymphedema after surgery and to decrease the patient’s reliance on Complex Decongestive Therapy (CDT) (22, 24).

Collectively, our findings demonstrate the safety and therapeutic efficacy of combined VLNT and liposuction in managing advanced bilateral LEL. Nevertheless, the absence of standardized, evidence-based systemic treatment protocols for bilateral lymphedema remains a significant clinical challenge. Given the heterogeneous pathophysiological manifestations across disease stages, treatment strategies require individualized approaches tailored to limb-specific severity. The development of comprehensive treatment algorithms necessitates further multicenter studies with larger patient cohorts and extended longitudinal follow-up.

## Data Availability

The original contributions presented in the study are included in the article/supplementary material. Further inquiries can be directed to the corresponding author.
